# Treatment of Inflammation of the Middle Ear

**Published:** 1892-12-03

**Authors:** 


					CENTRAL LONDON" HOSPITAL.
Treatment op Inflammation op the Middle Ear.
We must now return to the consideration of the treat-
ment of the different forms of middle-ear inflammation
mentioned in the first portion of this article in our issue
of November 26th.
I.?Acute Catarrh, Suppurative and Nonsuppurative.
?These two .forms will be dealt with together, as they
can hardly be distinguished in practice, the latter being
only a severe variety of the former. The milder kind
occurs commonly in children, and is especially asso-
ciated with adenoid vegetations, the cure of which, as
described above, generally completely stops the recur-
rent attacks; and if any deafness be subsequently left,
it must be treated by inflation of the middle ear for a
time, by one of the methods described at the end of thia
article.
The severe form requires more energetic treatment.
If the tympanic membrane be tense on account of fluid
within, it is incised. In order to perform this operation,
the membrane is made anaesthetic, as far as possible,
by applying to it a 10 per cent, solution of cocaine, on
a pledget of cotton wool; the largest sized speculum is
then used, and held in the left hand, and the opening
made in the posterior inferior quadrant of the mem-
brane with a small, sharply-pointed knife. It is
advisable that assistance should be handy to prevent
the patient moving at a critical moment. After an
opening has been made, a piece of cotton wool must be
kept in the ear for a time.
Most commonly, however, the patients do not come
to the hospital until the membrane has already been
perforated, and thus relief afforded by the severity of
the disease itself. The pain in some of these cases is
very severe, and to overcome this two or three leeches
are often applied to the mastoid, or in front of the
tragus, and also warmth may be applied by means of a
cloth wrung out of hot water; but poultices are not used.
At the same time, instillations, consisting of morphia'
about two grains to the ounce; and cocaine, about 10
per cent,, may be put into the ear. It may here be
mentioned that in using ear drops, these should first be
warmed, and this is best done by heating a spoon in
hot water, and then pouring the lotion into this before
use. When the acute symptoms have subsided, the
subsequent treatment consists in keeping the ear clean
by syringing it out with a lotion such as boracic acid
(gr. x. ad ?j.)? and daily inflation of the middle ear for
a time by Poltizer's method (vide infra). Should the
inflammation spread to the mastoid, as indicated by
oedema and pain in this situation, an incision is made
down to the bone, and it is sometimes found necessary
even to open the mastoid cells, but we Bhall speak of this
again later.
II.?Chronic Suppurative Catarrh.?This is, we sup-
pose, the commonest form of middle ear inflammation
which has to be treated at an Ear Hospital. The acute
attack of inflammation, of which it is the remnant, in
the majority of cases, has often been quite unnoticed
during the height of one of the exanthemata, such as
measles or scarlet fever, which has caused it. The
patient is then brought for treatment for a chronic
discharge of pus from the ears. In treating these
cases, the two points which are always kept in view
are cleanliness and removing everything which would
in any way hinder the free escape of the pus. In order
to keep the ear clear, some alkaline lotion is ordered,
and the patient directed to syringe the ear with this
two or three times daily. The lotions in common use
are Lotio Glauberi, which consists merely of sodium
sulphate ^ij. to water gx., or a lotion of bicarbonate of
soda gr. 100, acidii carbolici 5ij., aqua 5X. The
great advantage of the alkali is that it dissolves
anything of the nature of cerumen, &c., which
may be present. In addition to this cleansing,
instillations are applied, such as one consisting of
boracic acid, gr. xvi., water 3]"; or, if an astringent is to
be used, solution of lead Bubacetate 313, water giij; or
again, zinc sulphate, gr. v., water gj. As the case pro-
gresses, inflation of the middle ear must be undertaken
to prevent ankylosis of ossicles and adhesion of the
membrana tympani to the inner wall of the tympanum.
If the perforation in the drum be large and do not close
up, it is uaual to try if one of the forms of artificial
membrane will assist the hearing. Those most com-
monly tried are painting the remnants of drum with
collodion, or inserting a drop of glycerine, or placing a
pledget of wet cotton wool in contact with the ossicles.
Whether any of these methods will do any good can
only be found by trial.
A few words must now be said as to the treatment of
complications which may arise in the course of these
cases. The most important of these is the spread of
the inflammatiou to the mastoid cells, as evidenced by
pain, tenderness and oedema over that process of bone,
and perhaps the formation of pus superficial to it.
This last may occur without the cells themselves being
involved, in which case all that is required is an in-
cision to let out the pus. If the bone itself be involved,
this is exposed by turning back a flap of skin, and then
a free opening is made into the cells by means of the
Bmall trephine used for this purpose, or more often by
carefully chipping away the outer wall with a small
sharp gouge. After an opening has been made the pus is
washed out with such antiseptic lotions as biniodide
of mercury (red iodide of mercury gr. J, sodium iodide
gr. J, water 3j.), or corrosive sublimate (1 to 2,000).
Care is taken to see that the lotion can be syringed
right through from the mastoid and out through the
external meatus. Drainage tubes are then pat in, and
the ear frequently syringed; the tubes are kept in until
the discharge has practically ceased, and then the case
is treated in the ordinary way. Other complications
which may occur are thrombosis of the lateral sinus,
meningitis, and cerebral abscess. The last, of course,
Dec. 3, 1892. 7HE HOSPITAL. 155
if it is discovered, must be treated by letting out the
pus, the other two on general principles.
The constitutional treatment of middle ear sup-
puration consists in improving the general condition
by hygienic measures, and such drugs as cod liver oil
and the like.
III.?Chronic Nonsuppurative Catarrh.?One of the
most important features of this trouble is the stenosis
of the Eustachian tube, with which it is commonly
associated; and in order to overcome ithis, which by
preventing communication of the middle ear and the
pharynx causes much deafness, inflation is practised
either by means of a Politzer's bag or by passage of the
Eustachian catheter if it cannot be done otherwise. This,
at the same time, assists in preventing the fixation of
the ossicles, which would he caused by the chronic in-
flammatory thickening of the mucouB membrane sur-
rounding them. At the same time by these means
Vapour of Benzol (benzol inxl., oil of cassia m ij., light
magnesian carbonate gr. xx., and water ?1. One
drachm of this to a pint of hot water may be blown
into the middle ear by Politzer's bag. It may also be
done by the self-inflator, or Yalsolva's method. This
inflation of the ear should not be kept up daily for a
longer time than a month, and then a watch must be
kept on the tympanic membrane, to see that it does not
get too relaxed.
The various methods of inflating the ear must now
be shortly described.
Politzer's method consists in making the patient shut
off his pharynx from his naso-pharynx, by either
swallowing a mouthful of water, or blowing out his
cheeks, or pronouncing the syllable " hue." Then at
this moment, by means of a Politzer's bag, which has
a teat-like indiarubber end (see diagram) which fits
into one nostril, the other being firmly closed with the
finger and thumb, a blast of air is blown in. If only
one ear is to be acted on, the sound external meatus
must be closed with the finger. Sometimes the bag is
made with a long rubber tube attached to the nozzle,
so that the patient can use it himself ; and again,
there may be an enlargement at ecme point of the
tube, into which any medicated substance which it is
wished to blow into the middle ear can be placed, such
as Tinct. Chloroformi, so often prescribed in chronic
catarrh.
In Valsalva's method the patient closes his own nostrils
and mouth, and then forcibly attempts to blow through
his nose. If it is wished by this means to apply a
vapour, the air passages must first be filled with the
Vapour by inhalation. Another way is to employ the
self infiator used at the Central London, the medicated
substance being put on cotton wool, in the bulb near
the mouth piece (see diagram), and then the patient
having put the nose piece in position, and closed the
nostrils tight round it, blows vigorously through the
tube with the mouth.
To pass the Eustachian catheter, hold it with the point
directed downwards, and pass it along the floor of the
nose, till it reaches the posterior wall of the pharynx,
then draw it gently forward until it commences to catch
against the nasal floor; now by a half turn, and at the
Bame time carrying the outer end a little to the opposite
side, the point will usually, after one or two trials, be-
come engaged in the Eustachian orifice.
if it is discovered, must be treated by letting out the substance being put on cotton wool, in the bulb near
pus, the other two on general principles. the mouth piece (see diagram), and then the patient
The constitutional treatment of middle ear sup- having pat the nose piece in position, and closed the
puration consists in improving the general condition nostrils tight round it, blows vigorously through the
by hygienic measures, and such drugs as cod liver oil tube with the mouth. ^
and the like. To pass the Eustachian catheter, hold it with the point
III.?Chronic Nonsuppurative Catarrh.?One of the directed downwards, and pass it along the floor of the
most important features of this trouble is the stenosis nose, till it reaches the posterior wall of the pharynx,
of the Eustachian tube, with which it is commonly then draw it gently forward until it commences to catch
associated ; and in order to overcome ithis, which by against the nasal floor; now by a half turn, and at the
preventing communication of the middle ear and the same time carrying the outer end a little to the opposite
pharynx causes much deafness, inflation is practised Bide, the point will usually, after one or two trials, be-
either by means of a Politzer's bag or by passage of the come engaged in the Eustachian orifice.
Eustachian catheter if it cannot be done otherwise. This,
ST. MARY'S HOSPITAL.
The Treatment of Enteric Fever.
The treatment of enteric fever at St. Mary's will be
discussed under the following headings: (1) The
hygienic and dietetic treatment;
(2) the treatment by stimulants and
drugs ; (3) the treatment of the
pyrexia; (4) the treatment of the
complications.
1. The Hygienic and Dietetic Treatment.?The patient
is put to bed in a large and airy ward, the temperature
Potman's Bag. of which is maintained at about 60 deg. E. The bed
should be firm, and is so arranged that the patient can
at the same time, assists in preventing the fixation of be reached from either side. The coverings should be
the ossicles, which would be caused by the chronic in- light, but at the same time adequate, to protect the
flammatory thickening of the mucous membrane sur- patient from changes of temperature in the ward. The
rounding them. At the same time by these means patient is under no circumstances allowed to get out of
V apour of Benzol (benzol in xl., oil of cassia m i j., light bed, and the bed pan and urine bottle are used through-
magnesian carbonate gr. xx., and water gl. One out the whole attack, so that the recumbent position
drachm of this to a pint of hot water maybe blown is maintained, a point of considerable importance,
into the middle ear by Politzer's bag. It may also be The stools are examined daily by the house physician
done by the self-inflator, or Valsolva's method. This or sister of the ward, and are thoroughly disinfected
inflation of the ear should not be kept up daily for a before being thrown away. The patient is sponged
longer time than a month, and then a watch must be twice daily with cool or tepid water, to which vinegar
kept on the tympanic membrane, to see that it does not may or may not be added. The temperature of the
^rnv.00 re\axe<^" . water may be modified to suit the feelings of the
The various methods of inflating the ear must now patient. The sponging is carried out more fre-
?Portly described. ^ quently when circumstances, such as restlessness,
Jroutzer s method consists in making the patient shut &c., require it. The temperature is under ordinary
off his pharynx from his naso-pharynx, by either circumstances taken every four hours. The diet
swallowing a mouthful of water, or blowing out his consists exclusively of liquids, and is given as a rule in
cheeks, or pronouncing the syllable " hue." Then at the form of milk and beef tea. Two to three pints of
this moment, by means of a Politzer's bag, which has milk and one to one and a half pints of beef tea are
a teat-like indiarubber end (see diagram) which fits given in the twenty-four hours, and this amount is
into one nostril, the other being firmly closed with the rarely exceeded. The milk and beef-tea are given
linger and thumb, a blast of air is blown in. If only alternately at intervals of two or three hours, ac-
cording to the discretion of the nurse
with regard to disturbing the patient.
severe cases and in emergencies, the
patient is fed much more frequently, and
the food may have to be given in much
more concentrated form. In all cases it
is of importance that an exact record
should be kept of all foods taken by the
patient. The stools are examined daily
for curds, and if these are present, some
bilf-insufflatob. substance such as lime water, carbonate of soda, or
one ear is to be acted on, the sound external meatus seltzer is added to the milk. In many cases it is sufficient
must be^ closed with the finger. Sometimes the bag is to reduce the quantity of milk taken. Should the beef
made with a long rubber tube attached to the nozzle, tea excite diarrhoea, as is fairly commonly found to be
so that the patient can use it himself ; and again, the case, some substitute such as one of the many pre-
there may be an enlargement at Bome point of the parations of meat extracts or meat jellies may be tried,
tube, into which any medicated substance which it is The patient is allowed to drink freely of cold water,
wished to blow into the middle ear can be placed, such toast water, barley water, or any bland fluid in order
as Tinct. Chloroformi, so often prescribed in chronic to assuage his thirst. Small lumps of ice may be
catarrh. sucked for the same purpose. In those cases in wnich
In Valsalva's method the patient closes his own nostrils the milk, given with the precautions above mentioned,
and mouth, and then forcibly attempts to blow through cannot be digested, it may be tried in the peptonised
his nose. If it is wished by this means to apply a form, or Nestle's food may be given. The greatest
vapour, the air passages must first be filled with the care is observed during early convalescence with regard
Vapour by inhalation. Another way is to employ the to the resumption of solid food. It is usual at St.
self inflator used at the Central London, the medicated Mary's to wait at least seven days after the tempera.

				

## Figures and Tables

**Figure f1:**
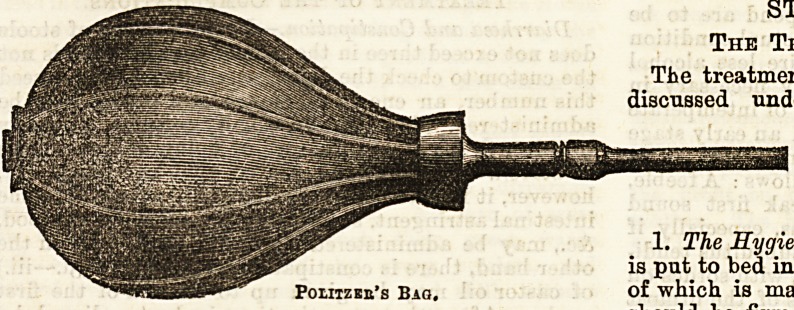
Politzer's Bag

**Figure f2:**
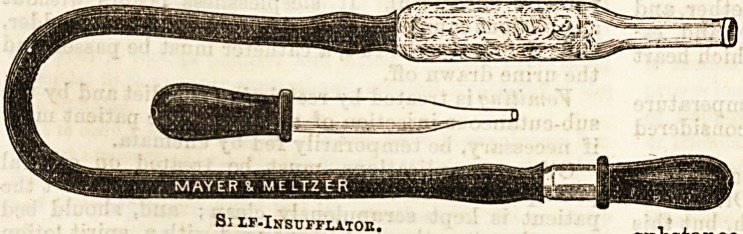
Self-Insufflator

